# Surname match/mismatch revisited: It may no longer matter for partner choice

**DOI:** 10.1371/journal.pone.0343333

**Published:** 2026-03-25

**Authors:** Takefumi Nakazawa, Naoto Shinohara, Yuya Fukano

**Affiliations:** 1 Department of Life Sciences, National Cheng Kung University, Tainan, Taiwan; 2 Center for Ecological Research, Kyoto University, Otsu, Japan; 3 Quantitative macroecology group, Okinawa Institute of Science and Technology Graduate University, Onna, Japan; 4 Faculty of Horticulture, Chiba University, Matsudo, Japan; University of Washington, UNITED STATES OF AMERICA

## Abstract

Kin recognition plays a crucial role in mating across various biological taxa, including bacteria, plants, and animals, to prevent inbreeding. Historically, surnames have been indicators of kinship in humans, with many countries enforcing laws and norms to prohibit marriages between individuals sharing the same surname in the past. Here, considering the potentially adaptive role of surname mismatch in inbreeding avoidance, we address whether surnames can still influence partner choice in modern human society, where same-surname marriages are no longer prohibited, by utilizing nation-wide name databases from Taiwan. Analysis of data from 2018 revealed a lower frequency of same-surname marriages than expected, reflecting historical taboos against such marriages. However, this pattern was not observed in the 2023 data, suggesting a potential shift in people’s attitudes over time. To further interpret this trend, we conducted a simple questionnaire survey among university students, which showed no significant correlation between surname coincidence and attractiveness ratings of heterosexual face images, in contrast to the traditional custom. Our results, although preliminary, suggest the hypothesis that the significance of surname match/mismatch in partner choice may have diminished, potentially due to rapidly changing socio-cultural environments. Further research into the shifting role of surnames is necessary to gain a deeper understanding of human social behaviors across different regions and contexts amidst modernization and globalization.

## Introduction

Kin recognition is widely observed across diverse biological taxa, including bacteria, plants, and animals, playing a crucial role in mating systems [1–3]. In humans, inbreeding depression poses significant health risks, making the adoption of inbreeding avoidance advantageous [4]. To regulate mating behavior conditionally in response to kinship in large society, it is crucial to estimate the kinship of the potential mating partner [5]. Humans have developed various kin recognition and socio-cultural systems to avoid inbreeding or endogamy due to evolutionary adaptation and/or cultural accommodation, based on phenotypic cues (e.g., olfaction and face [6,7]), environmental cues (e.g., coresidence durations in childhoods [8]), and social norms (e.g., incest taboo [9,10]).

The hereditary surname is found in many cultures around the world. Surnames, like DNA of organisms, can be passed down from generation to generation, and thus surnames have often been used in genetic studies (so-called isonymy) [11–13]. The history of surnames differs significantly between Europe and Asia, shaped by distinct cultural, social, and historical contexts [14]. While the use of surnames became common during the Middle Ages (e.g., around the 11th to 15th centuries) in Europe [15], it has a much earlier origin in Asia. In China, for example, surnames were already in use by around 2000 BCE, and they were typically passed down through the paternal line, often linked to family heritage, clan identity, and social status [16]. The use of surnames, although relatively recent in the context of human evolutionary history, may have a significant influence on partner choice. In times when individual dispersal was more limited than it is today, a surname match would have been a clear indication of kinship, sharing a close ancestor. Consequently, individuals sharing the same surname were likely to be avoided as potential mating partners to prevent inbreeding in the past, although the kinship-signaling function of surnames has diminished in adaptive significance in modern societies characterized by widespread geographic mobility.

Such surname-based partner choice has played an important functional role in many Asian societies, in contrast to most Western societies, where surnames are only weakly associated with genetic relatedness [17–19] and normative prohibitions against same-surname marriages have been minimal or absent. In several Asian societies, surnames are more strongly tied to patrilineal kinship [20,21], and strong normative prohibitions against same-surname marriages have historically existed. For example, there were laws and norms that prohibited marriages with individuals sharing the same surname in such countries and regions, such as Korea [22], China [23], Aborigines in Australia [24], Kachin in imperial Vietnam [25], and Toba Batak in Indonesia [26]. In modern societies in these Asian countries, however, same-surname marriages are no longer prohibited. In China, the prohibition on marriage between individuals with the same surname began during the Tang Dynasty (618–907 CE) but the relevant laws were officially abolished in the early 20th century [27,28]. Nevertheless, such marriage has been avoided afterward due to cultural and traditional beliefs in many areas of China, likely because the long-standing legal prohibitions became entrenched as social values and norm, reflecting the continued influence of kinship norms on marriage practices [29]. In Korea, a clan-based law prohibiting marriages between individuals with the same surname and ancestral origin was only recently abolished in the early 2000s [30]. Despite the cultural significance of surnames, their role in marriage patterns in modern Asian societies remains inadequately studied.

Here, we focus on modern Taiwanese society to examine the impact of surnames on partner choice. Since the 17th century, many Han Chinese migrated from mainland China to Taiwan. These immigrants primarily came from Fujian and Guangdong provinces, bringing their culture and customs with them. In addition to these early migrations, a substantially larger number of migrants arrived after World War II, profoundly reshaping Taiwan’s demographic structure [31]. In China, marriage between individuals sharing the same surname was legally prohibited until the early 20th century (see above). These prohibitions were strictly enforced only a few generations ago and thus form part of the lived experiences of older generations in Taiwan, either directly or through familial transmission. Such historical regulations were embedded within broader Confucian principles that emphasize respect for family lineage, elders, and social order that have been widely internalized across East Asian societies (i.e., filial piety). These Confucian principles have long shaped behavioral norms in East Asia in ways that differ from those in Western societies [32,33]. The persistence of same-surname marriage avoidance in East Asia is further illustrated by Korea, where such marriages were prohibited through codified legal restrictions until the early 2000s [30]. In contrast, Taiwan has never imposed formal legal restrictions on same-surname marriages. Nevertheless, the practice has been traditionally avoided and appears to be maintained primarily through customary norms rather than law, and prohibitions on same-surname marriages that were legally enforced in China until relatively recently may continue to influence contemporary marital practices in Taiwan, even in the absence of explicit legal constraints. Notably, surname diversity is low and the distribution of surnames is uneven in Taiwan, with certain surnames being extremely common (S1 File), due to the historical migration of Chinese settlers [34], leading to the situation that individuals frequently encounter those sharing the same surname. Moreover, married couples in Taiwan typically retain separate surnames, and the government has maintained nationwide databases of married surnames, publishing parts of these databases every few years. These features of Taiwanese society provide an opportunity to test, at a population scale, whether social norms that may have originated from the biologically adaptive significance of avoiding inbreeding can still influence partner choice in modern society. Utilizing the published data, we aim to test for the first time whether modern individuals still tend to avoid marrying those with the same surname on a national scale. Additionally, to experimentally elucidate behavioral mechanisms, we conducted a simple questionnaire survey. We hypothesized that respondents would rate the attractiveness of faces with the same surname lower than those with different surnames if same-surname marriages are avoided.

## Materials and methods

### Name databases

The Ministry of the Interior in Taiwan has published a booklet entitled “Name Statistics” several times in the past decades, which provides various statistical information on names in Taiwan. We utilized the two most recent versions of this booklet published in 2018 and 2023 [35,36], because they contain information on ten major combinations of surnames in marriages separately for same-surname and different-surname couples. (S2 File). In the 2018 data, the total number of marriages was 5,353,385, of which 175,933 couples had the same surnames [35]. In the 2023 data, the total number of marriages was 5,231,570, of which 226,871 couples had the same surnames [36]. Therefore, the frequency of same-surname marriages has increased between the two years from 3.29% in 2018 to 4.34% in 2023, contrary to historical taboos against such marriages. Unfortunately, earlier versions of the booklet did not include the information on combinations of surnames in marriages. Taiwan has many Indigenous peoples. However, their total population accounts for less than 3% of the national population [35,36]. Therefore, whether or not their ethnic backgrounds are considered is unlikely to critically affect results of the name database analysis, even if they have surnames characteristic of certain Indigenous peoples (see also Discussion). We also note that the information on less frequent combinations of surnames in marriages was withdrawn due to privacy concerns despite our repeated requests for data openness to the government. As such, our analysis in the present study focused on ten major combinations of same-surname and different-surname couples in marriages. When considering only the ten major combinations, the numbers of same-surname and different-surname marriages were 141,370 (ca. 80.4% of same-surname marriages) and 517,379 (ca. 9.7% of all marriages), respectively, in 2018, and 182,800 (ca. 80.6% of same-surname marriages) and 558,451 (ca. 10.7% of all marriages), respectively, in 2023.

First, we calculated the expected frequencies of marriages between the same (or different) surnames based on the frequencies of the ten major surnames in the population and their random combinations. For example, in 2018, 11.2% and 11.1% of Taiwanese males and females, respectively, have the surname “Chen” (S1 File). Therefore, the expected frequency of marriages between male “Chen” and female “Chen” is calculated as 11.2% × 11.1% = 1.24%. Similarly, we calculated the expected frequencies of the ten major combinations for different-surname marriages. For example, the expected frequency of “Chen-Lin” marriages is calculated as (the frequency of “Chen” in males) × (the frequency of “Lin” in females) + (the frequency of “Lin” in males) × (the frequency of “Chen” in females). We then examined whether the expected frequencies of same-surname (or different-surname) marriages were lower (or higher) than the observed frequencies in the 2018 and 2023 databases [35,36]. The difference between the expected and observed frequencies was tested using the binomial test. It is important to note that the observed frequencies of married surnames represent proportions among the entire married couples, including those who have been married in the past and are still married, but not proportions among those who married in those specific years. Thus, the present analysis cannot provide snapshots of people’s attitudes at any given time. Nevertheless, by comparing results between the two years, we can detect temporal changes in the influence of surnames on marriage. We also note that changing surnames is generally not permitted without family-related or legal justification under Taiwanese law, in contrast to some Western countries where surname changes are more easily allowed (see also Discussion). Therefore, we did not consider surname changes in our analysis of the name database. While this was not feasible due to data limitations, it was unlikely to critically affect our results, as the frequency of surname changes would be relatively low.

### Questionnaire survey

In addition to the database analysis of surname combinations in marriages (see above), we conducted a simple online questionnaire survey with Taiwanese students from National Cheng Kung University by using Google Forms (S3 File) in Mandarin during Jun 2022 (6/1/2022–6/24/2022). The university is a large national institution that attracts numerous students from all regions of Taiwan, and most of the respondents were students enrolled in our courses. We again did not consider their ethnic backgrounds because their small population size was unlikely to critically affect results of the questionnaire survey. We invited students to participate in the survey by sending requests to their university email accounts through the online course management system. A smaller portion of respondents consisted of students we encountered on campus, whom we invited to participate in person after confirming their enrolment and Taiwanese nationality. Although we did not have precise control over their ages, the participants appeared to be around their early twenties. We also acknowledge a potential sampling bias resulting from recruiting within a single university (see [37,38] for discussion of the so-called WEIRD bias). Nevertheless, this sampling method remains common in psychological research and can be useful for interpreting the database analysis in Taiwan and for identifying novel phenomena that warrant more rigorous investigation in the future.

Respondents were prompted to indicate their biological genders and surnames, and to rate the attractiveness of 10 homo- and hetero-sexual face images on a scale from one (the lowest) to ten (the highest). To generate these images, we utilized AI-generated faces from Generated Photos [39], specifying Ethnicity (Asian), Face (natural), Head pose (front-facing), Age (young adult), Hair color (black), Hair length (short for males and medium for females), and Emotion (neutral). The quality of the AI-generated facial images was high (see S3 File), and no respondents exhibited reactions resembling the so-called “uncanny valley” effect. We randomly selected ten Taiwanese-looking face images for each gender, based on the consensus of two Taiwanese assistants. These face images were then labelled with the ten major surnames and given names specified from Name Statistics of Taiwan (S1 File). Decision-making processes of marriage or partner choice are complicated, determined by multiple factors (e.g., social and economic status), not just appearance. To eliminate confounding effects of other factors, we attempted to increase the sample size as large as possible while considering random effects in data analysis (see below). Finally, we gathered responses from 956 participants (478 males and 478 females), after filtering out inappropriate responses such as foreign surnames and English spellings. Among them, about 55% had the ten major surnames (278 males and 256 females).

We then examined whether respondents rated the attractiveness of face images differently based on whether the face images shared the same surname with respondents. We employed multiple regressions utilizing linear mixed models for analysis. The response variable was the attractiveness score assigned to each face image by each respondent. We constructed a generalized linear mixed-effects model to examine the effects of face image gender and surname coincidence on participants’ responses. The fixed effects included (i) the gender of the face images (categorized as “male” or “female”, with “female” set as the reference), (ii) the coincidence of surnames between the respondent and the face image (classified as “same” or “different”, with “different” set as the reference), and (iii) the interaction between gender and surname coincidence. We included random intercepts for both face image identity (10 male and 10 female 1iamges) and respondent identity (478 male and 478 female respondents) to account for repeated measures. Each respondent rated 10 male and 10 female face images, and each face image was evaluated by multiple respondents, resulting in a fully crossed random-effects structure. No random slopes were included in the model. Separate models were developed for male and female respondents. Although some images had relatively high or low scores compared to others, it does not matter in the present study because we were interested in effects of surname match/mismatch. All statistical analyses were conducted using R [40] and the lmerTest package [41]. See S4 and S5 Files for analytical data and code, respectively.

### Ethics statement

The online questionnaire was anonymous without requesting given names and conducted with the approval of National Cheng Kung University Human Research Ethics Committee. We also informed the participants of the survey guidelines and obtained their consent. All methods were carried out in accordance with relevant guidelines and regulations.

### Inclusivity in global research

Additional information regarding the ethical, cultural, and scientific considerations specific to inclusivity in global research is included in S6 File.

## Results

**Fig 1:**
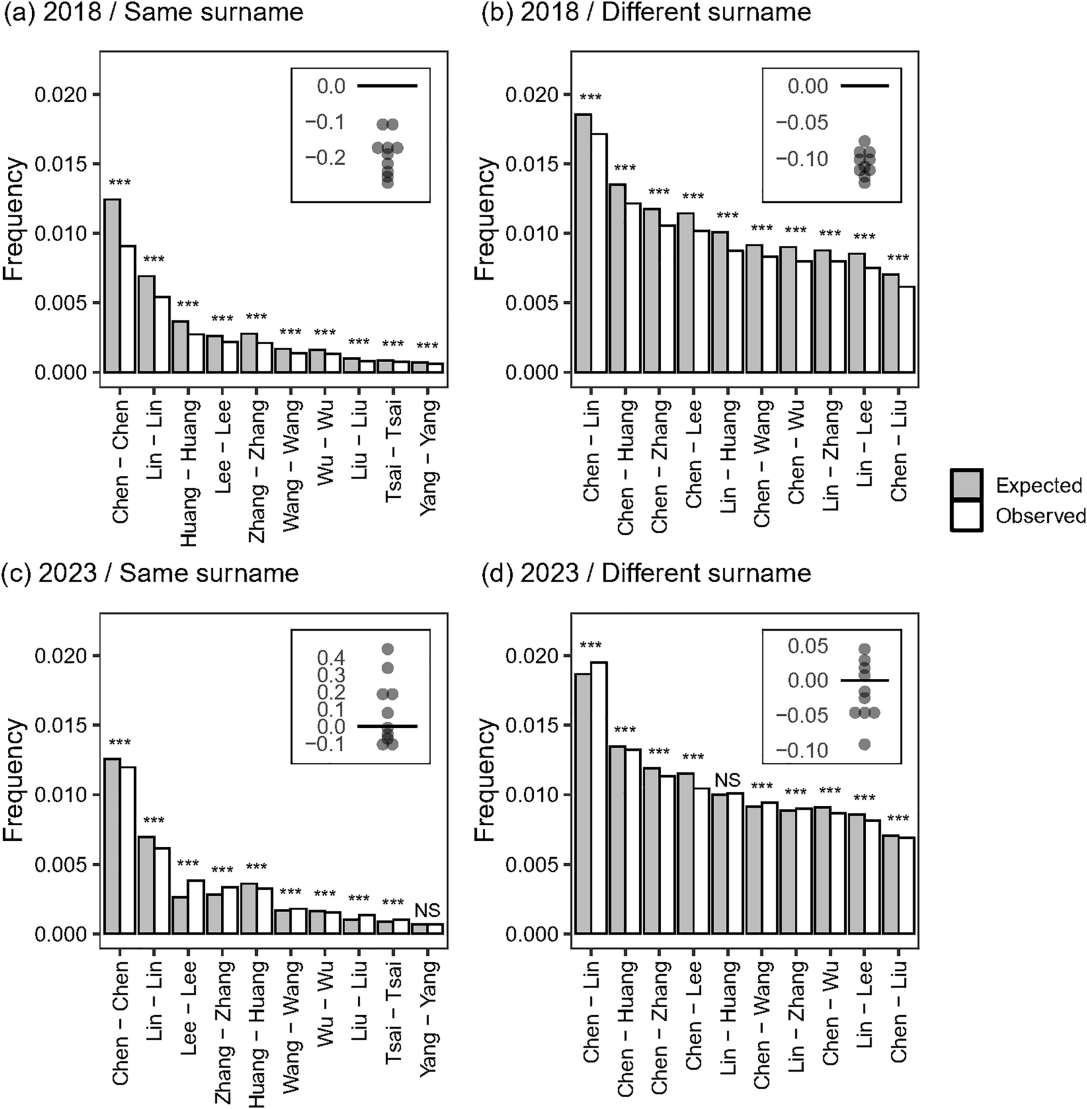
Results of the name database analysis. The datasets were published in (a, b) 2018 and (c, d) 2023. Males and females have (a, c) the same (b, d) different surnames. The grey and white bars indicate expected and observed frequencies of surname combinations. The inserts in the upper right corner represent the distributions of the standardized difference between the observed and expected frequencies (i.e., [observed-expected]/expected), where negatives values indicate that observed frequencies were lower than expected. The significance of the comparison between the observed and expected frequencies is also shown (***: *p* < 0.001; **: *p* < 0.01; *: *p* < 0.05; NS: p > 0.05).

In the 2018 data, the observed frequencies of the same-surname marriages were significantly lower than expected (*p* < 0.001 for all comparisons, Fig 1a). For example, the observed frequency of “Chen-Chen” marriages was 0.91% whereas their expected frequency was 1.24%. Notably, this was consistent for all of the ten major surnames. Meanwhile, we also found the same patterns for different-surname marriages and the observed frequencies were consistently lower than the expected frequencies, although seven out of the ten major combinations involved Chen (*p* < 0.001 for all comparisons, Fig 1b). For example, the observed frequency of “Chen-Lin” marriages was 1.71% whereas their expected frequency was 1.85%. In the 2023 data, however, such patterns were less consistent, with some pairs observed less frequently than expected (e.g., Chen–Chen), others more frequently (e.g., Lee–Lee), and one pair (i.e., Yang–Yang) showing no statistically significant difference in same-surname marriages (Fig 1c). A similarly inconsistent pattern was observed for different-surname marriages as well (Fig 1d).

**Fig 2:**
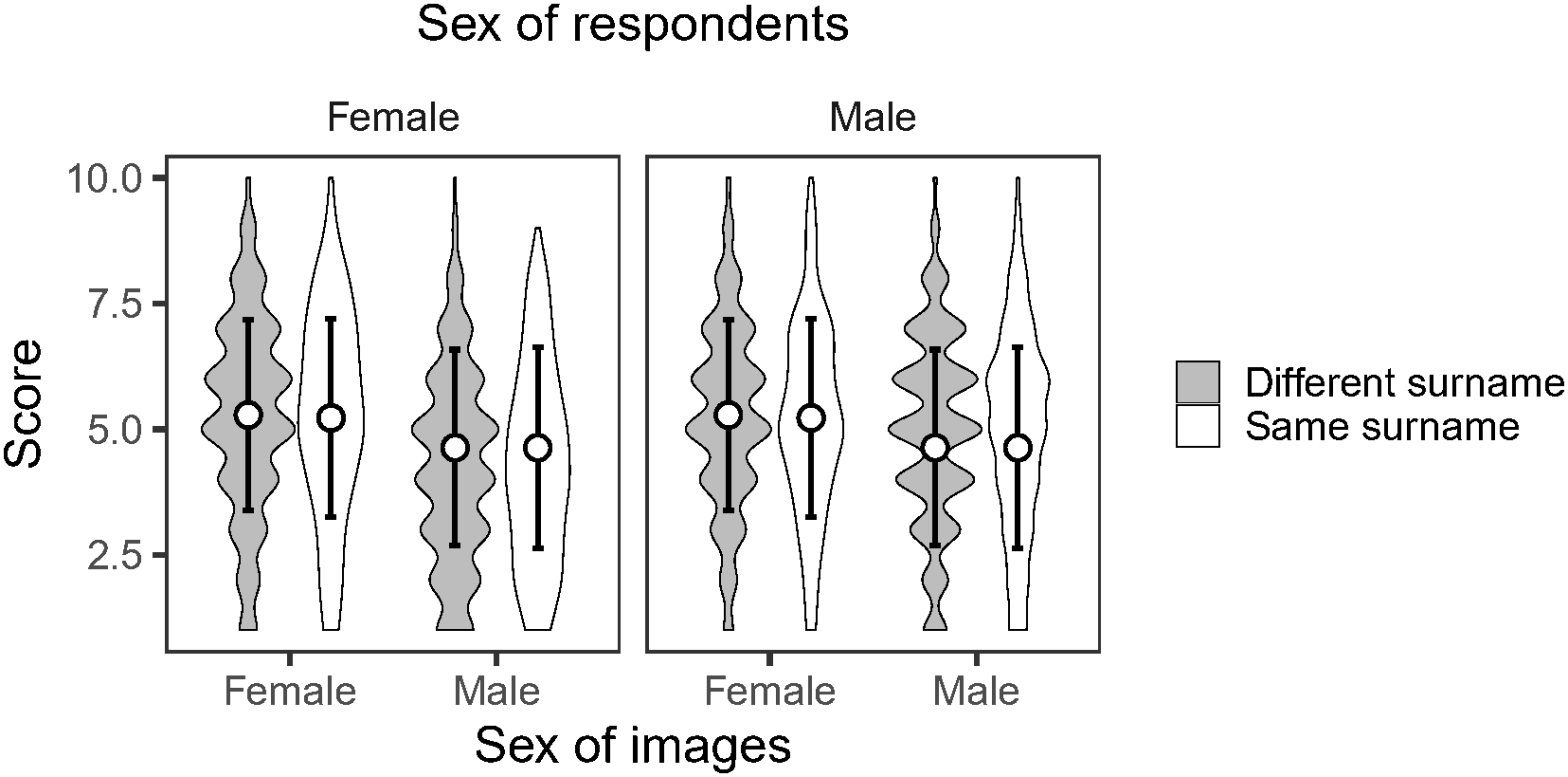
Results of the experimental questionnaire survey. The scores given by respondents are compared between the sex of face images and the coincidence of the surnames between respondents and face images. The means (open circles) and standard deviations (bars) are depicted with the violin plots. Respondents have either the same (grey) or different (while) surnames from face images.

In the experimental questionnaire survey, we found no significant interactive effect between the sex of face images and the surname coincidence between respondents and face images (Fig 2, Table 1, *p* = 0.806 for female respondents and *p* = 0.469 for male respondents). This result means that respondents gave the same scores to face images irrespective of whether the surnames were coincident with theirs.

**Table 1:** Results of the linear mixed models.

		Estimates (standard error)	*p*-values
Females	Sex of face images (Male)	−0.997 (0.254)	0.001
	Coincidence of surname (Same)	−0.037 (0.084)	0.661
	Sex (Male) × Surname (Same)	0.029 (0.118)	0.806
Males	Sex of face images (Male)	−0.305 (0.211)	0.166
	Coincidence of surname (Same)	−0.025 (0.078)	0.752
	Sex (Male) × Surname (Same)	0.079 (0.110)	0.469

The effects of the sex of face images (“female” is the reference therefore the effect of “male” is reported), the surname coincidence between face images and respondents (“different” is the reference therefore the effect of “same” is reported), and their interactive effects are tested by linear mixed models. The estimated coefficients, standard errors, and *p*-values are shown.

## Discussion

To the best of our knowledge, this study is among the first to suggest the possibility that surnames may influence partner choice, based on analyses of nationwide name databases. Analysis of the 2018 data revealed that same-surname marriages were significantly less common than expected, consistent with the hypothesis (Fig 1a). Historically, same-surname marriages have been a taboo in many countries and particularly Asian countries (see Introduction). In China, same-surname marriages were prohibited by law until the early 20th century [27,28]. The practice of avoiding the same-surname marriage in Taiwan is therefore considered to be one of the customs that Han Chinese immigrants brought from mainland China, particularly after World War II. Thereafter, the culture and family life have rapidly become freer and more open in Taiwan [42–44] (see also below). Our results suggest that despite socio-cultural changes in modern society, partner choice based on surname match/mismatch was still detectable at least until 2018.

We also found that different-surname marriages were also less common than expected in 2018, contrary to the hypothesis (Fig 1b). Therefore, surnames may have more complex effects on partner choice than initially hypothesized. Specifically, our results indicate that people tended to avoid marrying individuals not only with the same surname but also with major surnames (Fig 1b). In other words, people may have preferred different and relatively uncommon (or non-major) surnames for marriage. This finding may imply that a surname match serves as a clear indication of kinship for individuals with relatively uncommon surnames, whereas it may not be effective for identifying kinship among those with very common surnames. However, limitations in data accessibility prevented us from rigorously testing this idea in the present study. To more fully explore this possibility, an analysis of the entire database, including the undisclosed information on rarer surname combinations in marriages, is absolutely necessary in future work.

If people prefer non-major surnames for marriage, it may suggest that marriages with individuals with such surnames may bring certain social or economic benefits beyond the traditional norm of avoiding same-surname marriages. For example, if relatively uncommon surnames denote high social status or special skills as social elites [45], marriages with individuals having such surnames may contribute to increasing the power and wealth of the family from a perspective of social mobility [45–47]. Consistent with this idea, Hao [48] previously showed that by the 1990s in Taiwan, surnames characteristic of postwar immigrants were relatively overrepresented in higher education and high profile occupations compared with surnames characteristic of native Taiwanese whose families had settled in Taiwan before the war. His finding suggests that certain surnames may have been associated with social advantages several decades ago. Such patterns likely reflect the fact that Han Chinese immigrants to Taiwan after World War II mainly consisted of government and party personnel, military and military affiliated personnel, and intellectuals and professionals [31]. Here, it is important to note that Hao [48] focused on non-major surnames because major surnames, such as those we analyzed in the present study, were already widespread in both Taiwan and China and therefore could not distinguish postwar immigrants from native Taiwanese [48]. Taken together, these observations lead to the hypothesis that Taiwanese people may have preferred non-major surnames over major ones in marriage because of their potentially higher socioeconomic status.

Notably, we observed that the effects of surnames detected in the 2018 data were absent in the 2023 data (Figs 1c and 1d). We have two hypothetical explanations for this inconsistency. Firstly, our results may have been distorted to some degree as our data analyses were limited to the ten major surnames, although they accounted for more than 50% of the population in Taiwan (S1 File). That is, the patterns found in either 2018 and/or 2023 may have happened by chance due to the marginal effects. Validating this explanation requires a more extensive collection of longitudinal data, and future analyses are expected. The other possible explanation is that people’s attitudes may have actually changed over time, so a partner’s surname is no longer considered when marrying, unlike in the past. We consider that the second explanation is more plausible because Taiwanese people often express that surnames no longer hold significance in marriage although they did decades before (T. Nakazawa, simple interviews). Indeed, our questionnaire survey revealed that in the generation of college students, surnames had no impact on their assessment of heterosexual attractiveness (Fig 2). This explanation appears to broadly align with the previous finding showing that the association between surnames and socioeconomic status have largely disappeared by the 1990s as Taiwanese society became increasingly liberalized [48].

While we highlighted the potential influence of surnames in marriage or partner choice, demographic studies have reported that surnames may also play important roles in other social contexts. A classical study conducted in the United States in the 1940s found that, among college students who expressed dissatisfaction with their surnames, approximately two-thirds had non-Anglo-Saxon surnames, and most indicated a preference for selecting anglicized surnames [49]. This finding suggested that anglicized surnames could confer certain advantages in US society. More recent studies have shown that surnames are often closely associated with social or economic status in various countries, particularly in immigrant-friendly countries [50,51] and former colonial countries [52]. In such contexts, immigrants may even improve their income or occupational outcomes by changing their original surnames [53,54]. These studies demonstrate that changing surnames can serve as a mechanism for upward social mobility from a perspective of social mobility [45–47]. However, the situation differs in Taiwan, although the society was shaped by immigrants, because postwar immigrants tended to occupy higher social status [31,48]. In addition, in Taiwan, and likely in other East Asian countries as well, changing surnames is generally not permitted or is strictly regulated by law. Rather, maintaining surnames is considered important in East Asia, reflecting Confucian principles that emphasize respect for family, elders, and traditional customs [32,33], although adherence to these norms has been gradually declining. Consequently, marriage may provide a more plausible pathway for individuals or families to change their social strata in East Asian countries such as Taiwan. Studies on partner choice and mate preferences have largely been conducted within the framework of psychology in Western individualistic societies (see [55] for the influence of similarity in given names on partner choice). Here, we emphasize that the novelty of the present study lies in examining the role of surnames in intersexual contexts, such as marriage and partner choice, in East Asia, where conservative perspectives on family and marriage rooted in Confucian principles have been respected until relatively recently. Conducting a similar study in Korea would also be of interest because social norms avoiding same-surname marriages may still persist, as until the early 2000s, Korean law prohibited marriage between individuals with the same surname and ancestral origin [30].

Here, an important question arises. Can a five-year generational shift suffice to alter people’s attitudes? We consider that it is likely, given the rapid changes in socio-cultural norms in recent years in Taiwan [42–44,56,57]. For example, consider the Human Freedom Index (HFI), an international measure that combines personal and economic freedoms to assess the overall level of freedom in each country [58]. In 2000, Taiwan’s HFI ranked around 20th to 30th in the world, reflecting a moderate level of freedom. Since then, it has steadily increased, reaching the top 15 by the 2010s. In particular, significant improvements have been observed in personal and civil freedoms, making Taiwan one of the freest countries in Asia. In addition, the World Values Survey, a large-scale cross-national study tracking attitudes and values over time, provides insights into Taiwan’s social and individual freedom [59]. Since its first participation in 1995, Taiwanese respondents have shown a clear long-term increase in self-expression and personal autonomy values, reflecting greater emphasis on individual choice, freedom of opinion, and participation in social and political life. These trends indicate that, over the past decades, Taiwan has experienced significant cultural and social liberalization, complementing institutional and political reforms. An intriguing event in this context is the recent legalization of same-sex marriage in Taiwan in 2019 [57]. Such socio-cultural and environmental changes, or shifts in people’s attitudes, which likely occurred much earlier, may have influenced perspectives regarding the importance of surnames in marriage, particularly among young people in Taiwan, as shown by our questionnaire survey (Fig 2). In addition to the shifting perspectives on marriage among people, traditional views on marriage held by older generations would have also gradually disappeared from the name database due to generational turnover. The total number of married couples decreased by 121,815 from 5,353,385 in 2018 [35] to 5,231,570 in 2023 [36], despite marriages being more than twice as frequent as divorces each year [60]. This suggests that the number of spouse deaths is substantial and the impact of generational turnover may be considerable. To address the issue, a similar questionnaire survey could be conducted for older generations who may have traditional customs of avoiding marriage with individuals sharing the same surname. They might rate face images differently based on the coincidence of surnames with their own, unlike the present generation of college students. However, this approach lacks rigor because it cannot assess their partner preferences in the past when they married, considering recent changes in various socio-cultural environments that could have influenced their perspectives on marriage and family (see above). Another solution is to explore older databases of married surnames and track changes in the effects of surnames over time. However, this was also not feasible due to limited access to the government databases. In relation to temporal changes in population age structure, it is also important to note that Taiwan has experienced marked trends toward later marriage, declining fertility, and population aging over the past decades [61,62]. Under these circumstances, the influence of surnames in marriage could be underestimated in name databases, because younger generations, who are likely to hold more liberal norms regarding marriage, tend to delay marriage. This implies that our hypothetical argument (i.e., surname match/mismatch no longer matters in marriage) would be more plausible. We emphasize again the need for an analysis of the entire database, including non-major surname combinations in marriages as well as ages at which people married, across years.

Another important issue concerns the potential influence of geographical factors. Although the frequency distributions of major surnames seem largely similar across different regions in Taiwan, slight regional variations do exist [35,36]. While we examined the influence of surname match/mismatch on marriage at the national level, conducting similar analyses at the regional level would be of interest. Despite improvements in transportation and mobility in recent decades, individuals living in closer proximity may still have more opportunities to marry, a phenomenon known as the propinquity effect [63]. For example, Wu and Tsai [64] suggested that spatial contexts, such as schools and workplaces, can increase the likelihood of marriages in Taiwan. Therefore, in addition to age at marriage, geographical factors such as birthplace or current place of residence may also influence marriage patterns. We hypothesize that the influence of surnames may be weaker in more liberal urban or nearby suburban areas, and stronger in more conservative rural regions. Unfortunately, due to the lack of publicly available local-scale data on surname combinations in marriages, such analyses were not feasible in the present study. We propose this as an important direction for future research.

In the present study, we did not examine the interplay between surnames and ethnicity in the name database analysis and experimental questionnaire survey. Taiwan is a multi-ethnic country composed not only of Han Chinese but also of many Indigenous peoples, officially recognized as 16 tribes, who inhabited the Taiwan island long before the large-scale immigration of Han Chinese from mainland China beginning in the 17th century [65]. Indigenous peoples account for less than 3% of the total population [35,36]. Therefore, even if certain surnames were characteristic of particular Indigenous tribes, their small population size is unlikely to substantially affect our results. Rather, we note that their surnames would not be characteristic of particular Indigenous tribes, because during the Japanese colonial period (the early 20th century), when modern household registration systems were established, and later under the postwar Han Chinese–dominated government, Indigenous peoples were administratively assigned Chinese-style surnames. These assigned surnames were typically common and socially neutral Han Chinese surnames, potentially to avoid social disadvantages associated with ethnic identity [66]. Notably, with the 2024 revision of the “Name Act,” Indigenous peoples in Taiwan are now legally allowed to register and use their traditional Indigenous names (in their native language) on official documents. Traditional naming practices of Indigenous peoples in Taiwan were embedded in distinct cultural systems specific to each group and were structurally different from the Han Chinese patrilineal surname system [67]. Future studies integrating detailed ethnic identifies, regional information, and individual-level surname data would be desired to fully disentangle the roles of surnames, ethnicity, and social structure in marriage decisions in Taiwan and other multi-ethnic societies.

## Conclusions

By making every effort to fully utilize available data, we showed that the effects of surname match/mismatch on partner choice were detectable until recently (Figs 1a and 1b). Although the lack of data resolution remains a concern, our findings also suggest the possibility that surnames may no longer hold significance for partner choice (Figs 1c and 1d). This shift, if it really happened, could be attributed to historical changes in socio-cultural norms [42–44,56,57] and generational turnover, although the exact mechanisms remain unclear. Close cooperation and data disclosure by the government are needed to advance research on such a nationwide phenomenon. We also suggest that our hypothesis needs to be tested across different regions and contexts, because the present study focused on the nation-wide databases from Taiwan and Taiwanese student in a single university.

Shifting roles of surnames could be observed in other contexts as well as surname has long been considered to affect various individuals’ decision-making processes and social behaviors [68–72]. For example, Oates & Wilson [68] experimentally showed that when seeking help via email, individuals were more likely to receive cooperative responses from those sharing the same surname than from those with different surnames, suggesting that surnames can not only serve for inbreeding avoidance but also foster cooperation among blood relatives. With the modernization and globalization of human society, such efficacy of surnames may be changing. It is time to reconsider the contexts in which surnames still strongly influence or become less influential in individual decision-making for a better understanding of human social behaviors.

## Supporting information

S1 File Ten major surnames in 2018 and 2023. (XLSX)

S2 File Ten major combinations of surnames in same-surname and different-surname marriages in 2018 and 2023. (XLSX)

S3 File Format of online questionnaire survey. (DOCX)

S4 File Dataset of responses in online questionnaire survey. (XLSX)

S5 File R code for analyzing name statistics data. (DOCX)

S6 File Inclusivity-in-global-research-questionnaire. (DOCX)
