# Supplementary material for: Surname match/mismatch revisited: It may no longer matter for partner choice
Source: PLoS One. 2026 Mar 25;21(3):e0343333. doi: 10.1371/journal.pone.0343333 (PMC13016315; doi:10.1371/journal.pone.0343333)
Supplement: S3 File — (DOCX) [file pone.0343333.s003.docx]

**Supporting Information 3. Format of online questionnaire survey**

We conducted the following questionnaire by online in Chinese. The images are published under a CC BY license, with permission from Generated Photos (https://generated.photos/).

1. Please provide your surname and biological gender.
2. Please rate the attractiveness of the following ten men on a scale from 1 (lowest) to 10 (highest).


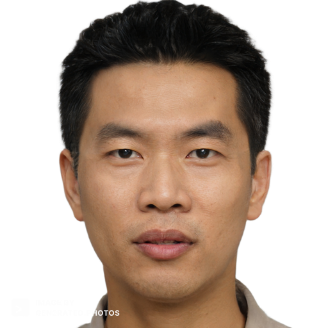

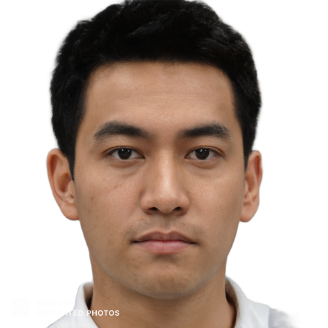

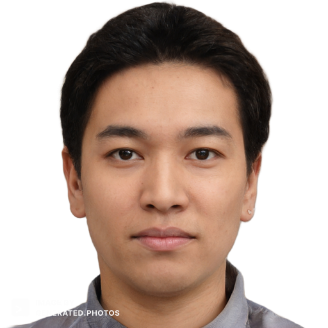

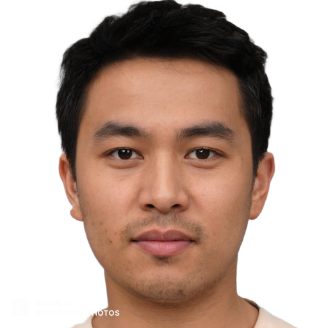

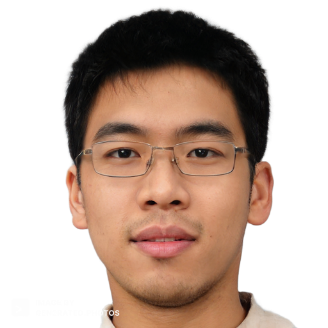


**陳柏翰**

**李俊傑**

**黃柏宇**

**蔡冠宇**

**劉宇翔**


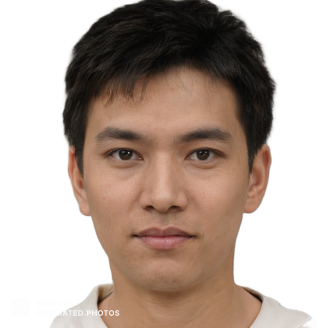

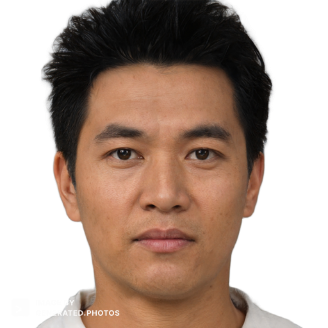

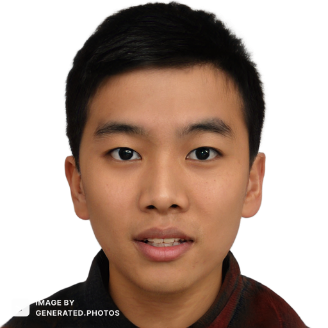

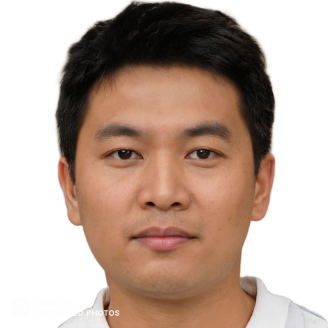

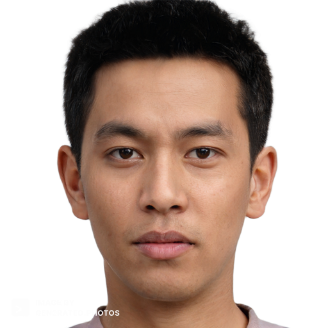


**楊冠霖**

**吳家豪**

**林承翰**

**張冠廷**

**王承恩**

1. Please rate the attractiveness of the following ten women on a scale from 1 (lowest) to 10 (highest).


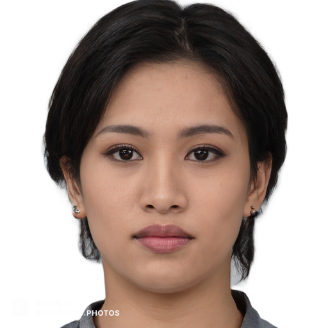

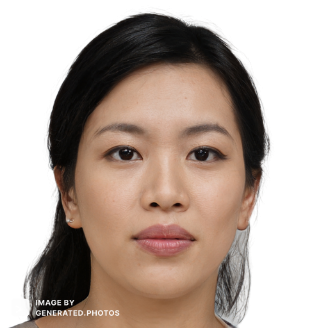

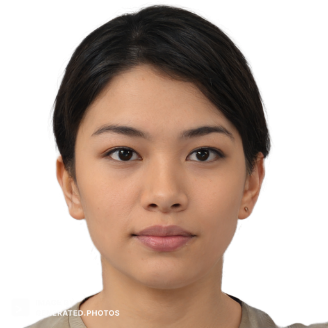

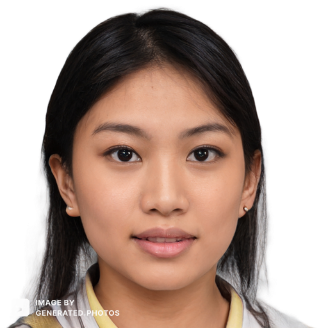

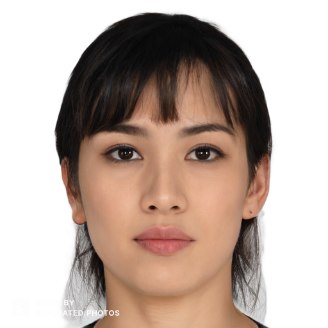


**黃宜蓁**

**蔡雅婷**

**王怡婷**

**張姿妤**

**劉欣妤**


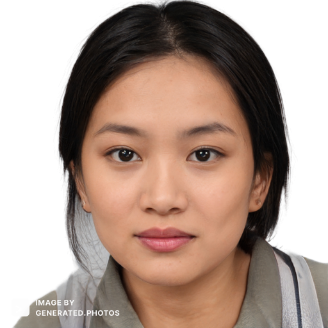

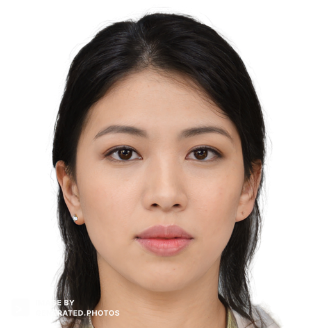

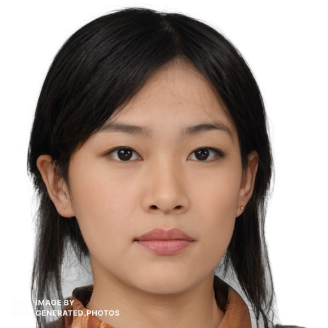

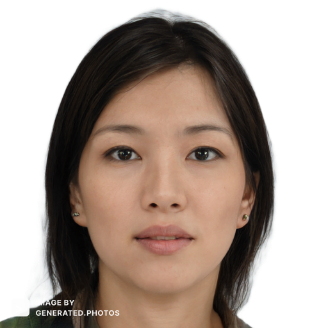

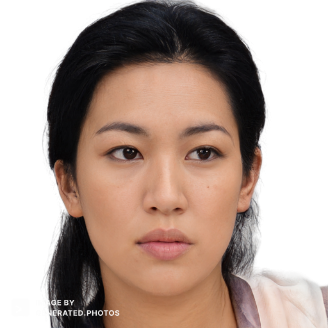


**楊思妤**

**陳欣怡**

**林怡君**

**吳怡萱**

**李詩涵**
