# Supplementary material for: Surname match/mismatch revisited: It may no longer matter for partner choice
Source: PLoS One. 2026 Mar 25;21(3):e0343333. doi: 10.1371/journal.pone.0343333 (PMC13016315; doi:10.1371/journal.pone.0343333)
Supplement: S5 File — (DOCX) [file pone.0343333.s005.docx]

**Supplementary Information 5**

Here, we provide the R code to analyse the name statistics data.

# Packages ----------------------------------------------------------------

library(tidyverse)

library(patchwork)

library(lmerTest)

library(gt)

# Read data ----------------------------------------------------------------

data_census_population <- read.csv("data_code_submission/data_census_population.csv")

data_census_couple <- read.csv("data_code_submission/data_census_couple.csv")

data_interview <- read.csv("data_code_submission/data_interview.csv")

# Note that in the data of the interview (data_interview), surnames of the respondents and images were replaced with dummy values (1:111)

# to protect the privacy of the respondents.

# But note that the 10 common surnames are labelled as 1:10.

# Top 10 common names ----------------------------------------------------------------

name_list <- tibble(name = c("陳", "林", "黃", "李", "張",

"王", "吳", "劉", "蔡", "楊"),

name_eng = c("Chen", "Lin", "Huang", "Lee", "Zhang",

"Wang", "Wu", "Liu", "Tsai", "Yang"))

# Arrange data ----------------------------------------------------------------

data_census_population_mod <- data_census_population %>%

mutate(freq_male = male/total_male,

freq_female = female/total_female)

data_census_couple_mod <- data_census_couple %>%

mutate(freq_couple = number_couple/total_couple) %>%

left_join(data_census_population_mod %>%

select(year, name_eng1 = name_eng, freq_male1 = freq_male, freq_female1 = freq_female),

by = join_by(year, name_eng1)) %>%

left_join(data_census_population_mod %>%

select(year, name_eng2 = name_eng, freq_male2 = freq_male, freq_female2 = freq_female),

by = join_by(year, name_eng2)) %>%

mutate(expected_freq_couple = case_when(name1 == name2 ~ freq_male1 * freq_female1,

name1 != name2 ~ freq_male1 * freq_female2 + freq_male2 * freq_female1))

# Basic information of interview data ----------------------------------------------------------------

# Number of same- and different-surname marriage in the database

data_census_couple_mod %>%

group_by(surname_match = ifelse(name_eng1 == name_eng2, "same", "different"), year) %>%

summarise(total = sum(number_couple)) %>%

pivot_wider(names_from = surname_match, values_from = total)

# Number of male and female respondents

data_interview %>%

distinct(id_respondent, .keep_all = T) %>%

group_by(sex_respondent) %>%

summarise(n = n())

# Number of respondents with common and uncommon surnames

data_interview %>%

distinct(id_respondent, .keep_all = T) %>%

mutate(commonness = if_else(name_key_respondent %in% as.character(1:10), "common", "uncommon")) %>%

group_by(sex_respondent, commonness) %>%

summarise(n = n())

# Example of calculating expected frequencies of couples in census data ----------------------------------------------------------------

# Same surname marriage: Chen - Chen

data_census_couple_mod[data_census_couple_mod$year == 2018 &

data_census_couple_mod$name_eng1 == "Chen" &

data_census_couple_mod$name_eng2 == "Chen", ]$freq_couple # Observed frequency

data_census_population_mod[data_census_population_mod$year == 2018 &

data_census_population_mod$name_eng == "Chen", ]$freq_male *

data_census_population_mod[data_census_population_mod$year == 2018 &

data_census_population_mod$name_eng == "Chen", ]$freq_female # Expected frequency

# Binomial tests of the difference between observed and expected frequencies ----------------------------------------------------------------

p <- c()

for(i in 1:nrow(data_census_couple_mod)){

binom_tmp <- binom.test(x = data_census_couple_mod$number_couple[i],

n = data_census_couple_mod$total_couple[i],

p = data_census_couple_mod$expected_freq_couple[i],

alternative = "two.sided")

p[i] <- binom_tmp$p.value

print(i)

}

# Fig. 1 ----------------------------------------------------------------

data_fig1 <- data_census_couple_mod %>%

bind_cols(p_binom = p) %>%

mutate(sig = case_when(p < 0.001 ~ "***",

p < 0.01 & p > 0.001 ~ "**",

p < 0.05 & p > 0.01 ~ "*",

.default = "NS"))

range_y <- c(0, 0.022)

## Fig. 1a ----------------------------------------------------------------

fig1a_main <- data_fig1 %>%

filter(year == 2018) %>%

filter(name1 == name2) %>%

pivot_longer(cols = c(freq_couple, expected_freq_couple),

names_to = "id", values_to = "freq") %>%

mutate(x = reorder(paste(name_eng1, name_eng2, sep = " - "), -number_couple)) %>%

ggplot() +

geom_bar(aes(x = x, y = freq, fill = id), stat = "identity", position = "dodge",

color = "black", linewidth = 0.5) +

geom_text(data = . %>%

group_by(x) %>%

filter(freq == max(freq)),

aes(x = x, y = freq + 0.001, label = sig)) +

scale_fill_manual(values = c("gray", "white"), labels = c("Expected", "Observed")) +

scale_y_continuous(limits = range_y) +

labs(y = "Frequency", subtitle = "(a) 2018 / Same surname") +

guides(fill = "none") +

theme_bw(base_size = 15) +

theme(panel.grid = element_blank(),

axis.text.x = element_text(angle = 90, hjust = 1, vjust = 0.5, color = "black"), axis.title.x = element_blank(),

axis.text.y = element_text(color = "black"), panel.background = element_rect(color = "black"),

plot.subtitle = element_text(hjust = -1.2, size = 15, vjust = 3),

legend.title = element_blank(), legend.position = c(0.8, 0.3), legend.background = element_blank()) ; fig1a_main

fig1a_insert <- data_fig1 %>%

filter(year == 2018) %>%

filter(name1 == name2) %>%

mutate(dif = (freq_couple - expected_freq_couple)/expected_freq_couple) %>%

ggplot() +

geom_dotplot(aes(x = 1, y = dif), binaxis = "y", stackdir = "center", dotsize = 3, alpha = 0.5) +

geom_hline(yintercept = 0) +

labs(y = "Expected ー Observed") +

theme_minimal(base_size = 15, base_family = "HiraKakuPro-W3") +

theme(panel.grid = element_blank(),

axis.title.x = element_blank(), axis.text.x = element_blank(),

axis.title.y = element_blank(),

plot.background = element_rect(colour = "black", linewidth = 0.5)) ; fig1a_insert

fig1a <- fig1a_main + inset_element(fig1a_insert, 0.55, 0.55, 0.95, 0.95) ; fig1a

## Fig. 1b ----------------------------------------------------------------

fig1b_main <- data_fig1 %>%

filter(year == 2018) %>%

filter(name1 != name2) %>%

pivot_longer(cols = c(freq_couple, expected_freq_couple),

names_to = "id", values_to = "freq") %>%

mutate(x = reorder(paste(name_eng1, name_eng2, sep = " - "), -number_couple)) %>%

ggplot() +

geom_bar(aes(x = x, y = freq, fill = id), stat = "identity", position = "dodge",

color = "black", linewidth = 0.5) +

geom_text(data = . %>%

group_by(x) %>%

filter(freq == max(freq)),

aes(x = x, y = freq + 0.001, label = sig)) +

scale_fill_manual(values = c("gray", "white"), labels = c("Expected", "Observed")) +

scale_y_continuous(limits = range_y) +

labs(y = "Frequency", subtitle = "(b) 2018 / Different surname") +

guides(fill = "none") +

theme_bw(base_size = 15) +

theme(panel.grid = element_blank(),

axis.text.x = element_text(angle = 90, hjust = 1, vjust = 0.5, color = "black"), axis.title.x = element_blank(),

axis.text.y = element_text(color = "black"), panel.background = element_rect(color = "black"),

plot.subtitle = element_text(hjust = -2, size = 15, vjust = 3),

legend.title = element_blank(), legend.position = c(0.8, 0.3), legend.background = element_blank()) ; fig1b_main

fig1b_insert <- data_fig1 %>%

filter(year == 2018) %>%

filter(name1 != name2) %>%

mutate(dif = (freq_couple - expected_freq_couple)/expected_freq_couple) %>%

ggplot() +

geom_dotplot(aes(x = 1, y = dif), binaxis = "y", stackdir = "center", dotsize = 3, alpha = 0.5) +

geom_hline(yintercept = 0) +

labs(y = "Expected ー Observed") +

theme_minimal(base_size = 15, base_family = "HiraKakuPro-W3") +

theme(panel.grid = element_blank(),

axis.title.x = element_blank(), axis.text.x = element_blank(),

axis.title.y = element_blank(),

plot.background = element_rect(colour = "black", linewidth = 0.5)) ; fig1b_insert

fig1b <- fig1b_main + inset_element(fig1b_insert, 0.55, 0.55, 0.95, 0.95) ; fig1b

## Fig. 1c ----------------------------------------------------------------

fig1c_main <- data_fig1 %>%

filter(year == 2023) %>%

filter(name1 == name2) %>%

pivot_longer(cols = c(freq_couple, expected_freq_couple),

names_to = "id", values_to = "freq") %>%

mutate(x = reorder(paste(name_eng1, name_eng2, sep = " - "), -number_couple)) %>%

ggplot() +

geom_bar(aes(x = x, y = freq, fill = id), stat = "identity", position = "dodge",

color = "black", linewidth = 0.5) +

geom_text(data = . %>%

group_by(x) %>%

filter(freq == max(freq)),

aes(x = x, y = freq + 0.001, label = sig)) +

scale_fill_manual(values = c("gray", "white"), labels = c("Expected", "Observed")) +

scale_y_continuous(limits = range_y) +

labs(y = "Frequency", subtitle = "(c) 2023 / Same surname") +

guides(fill = "none") +

theme_bw(base_size = 15) +

theme(panel.grid = element_blank(),

axis.text.x = element_text(angle = 90, hjust = 1, vjust = 0.5, color = "black"), axis.title.x = element_blank(),

axis.text.y = element_text(color = "black"), panel.background = element_rect(color = "black"),

plot.subtitle = element_text(hjust = -1.2, size = 15, vjust = 3),

legend.title = element_blank(), legend.position = c(0.8, 0.3), legend.background = element_blank()) ; fig1c_main

fig1c_insert <- data_fig1 %>%

filter(year == 2023) %>%

filter(name1 == name2) %>%

mutate(dif = (freq_couple - expected_freq_couple)/expected_freq_couple) %>%

ggplot() +

geom_dotplot(aes(x = 1, y = dif), binaxis = "y", stackdir = "center", dotsize = 3, alpha = 0.5) +

geom_hline(yintercept = 0) +

labs(y = "Expected ー Observed") +

theme_minimal(base_size = 15, base_family = "HiraKakuPro-W3") +

theme(panel.grid = element_blank(),

axis.title.x = element_blank(), axis.text.x = element_blank(),

axis.title.y = element_blank(),

plot.background = element_rect(colour = "black", linewidth = 0.5)) ; fig1c_insert

fig1c <- fig1c_main + inset_element(fig1c_insert, 0.55, 0.55, 0.95, 0.95) ; fig1c

## Fig. 1d ----------------------------------------------------------------

fig1d_main <- data_fig1 %>%

filter(year == 2023) %>%

filter(name1 != name2) %>%

pivot_longer(cols = c(freq_couple, expected_freq_couple),

names_to = "id", values_to = "freq") %>%

mutate(x = reorder(paste(name_eng1, name_eng2, sep = " - "), -number_couple)) %>%

ggplot() +

geom_bar(aes(x = x, y = freq, fill = id), stat = "identity", position = "dodge",

color = "black", linewidth = 0.5) +

geom_text(data = . %>%

group_by(x) %>%

filter(freq == max(freq)),

aes(x = x, y = freq + 0.001, label = sig)) +

scale_fill_manual(values = c("gray", "white"), labels = c("Expected", "Observed")) +

scale_y_continuous(limits = range_y) +

labs(y = "Frequency", subtitle = "(d) 2023 / Different surname") +

# guides(fill = "none") +

theme_bw(base_size = 15) +

theme(panel.grid = element_blank(),

axis.text.x = element_text(angle = 90, hjust = 1, vjust = 0.5, color = "black"), axis.title.x = element_blank(),

axis.text.y = element_text(color = "black"), panel.background = element_rect(color = "black"),

plot.subtitle = element_text(hjust = -2, size = 15, vjust = 3),

legend.title = element_blank(), legend.position = c(0.8, 0.3), legend.background = element_blank()) ; fig1d_main

fig1d_insert <- data_fig1 %>%

filter(year == 2023) %>%

filter(name1 != name2) %>%

mutate(dif = (freq_couple - expected_freq_couple)/expected_freq_couple) %>%

ggplot() +

geom_dotplot(aes(x = 1, y = dif), binaxis = "y", stackdir = "center", dotsize = 3, alpha = 0.5) +

geom_hline(yintercept = 0) +

labs(y = "Expected ー Observed") +

theme_minimal(base_size = 15, base_family = "HiraKakuPro-W3") +

theme(panel.grid = element_blank(),

axis.title.x = element_blank(), axis.text.x = element_blank(),

axis.title.y = element_blank(),

plot.background = element_rect(colour = "black", linewidth = 0.5)) ; fig1d_insert

fig1d <- fig1d_main + inset_element(fig1d_insert, 0.55, 0.55, 0.95, 0.95) ; fig1d

fig1a + fig1b + fig1c + fig1d + plot_layout(guides = "collect") # 800*800

# Fig. 2 ----------------------------------------------------------------

data_interview %>%

mutate(name_coin = if_else(name_key_respondent == name_key_image, "same", "different")) %>%

ggplot() +

geom_violin(aes(x = sex_image, y = score, fill = name_coin),

color = "black", linewidth = 0.2, position = position_dodge(width = 0.8)) +

geom_errorbar(data = . %>% group_by(name_coin, sex_image) %>%

summarise(mean = mean(score), sd = sd(score)),

aes(x = sex_image, ymin = mean-sd, ymax = mean+sd, group = name_coin),

position = position_dodge(width = 0.8), width = 0.1) +

geom_point(data = . %>% group_by(name_coin, sex_image) %>%

summarise(mean = mean(score)),

aes(x = sex_image, y = mean, group = name_coin),

shape = 21, size = 4, fill = "white", stroke = 1, position = position_dodge(width = 0.8)) +

scale_fill_manual(values = c("gray", "white"), labels = c("Different surname", "Same surname")) +

facet_wrap(~ sex_respondent, labeller = labeller(sex_respondent = c("female" = "Female", "male" = "Male"))) +

scale_x_discrete(labels = c("Female", "Male")) +

labs(x = "Sex of images", y = "Score", title = "Sex of respondents") +

theme_bw(base_size = 20) +

theme(panel.grid = element_blank(), strip.background = element_blank(),

plot.title = element_text(hjust = 0.5, size = 20), legend.title = element_blank()) # 900*450

# Table. 1 ----------------------------------------------------------------

lmm_female <- lmer(score ~ sex_image + name_coin + sex_image:name_coin + (1|id_image) + (1|id_respondent),

data = data_interview %>%

filter(sex_respondent == "female") %>%

mutate(name_coin = ifelse(name_key_respondent == name_key_image, "same", "different")))

lmm_male <- lmer(score ~ sex_image + name_coin + sex_image:name_coin + (1|id_image) + (1|id_respondent),

data = data_interview %>%

filter(sex_respondent == "male") %>%

mutate(name_coin = ifelse(name_key_respondent == name_key_image, "same", "different")))

summary_female <- summary(lmm_female)

summary_male <- summary(lmm_male)

lmm_res <- expand_grid(sex_student = c("Female", "Male"),

tibble(' ' = c("Sex of image (Male)",

"Coinsidence of surname (Same)",

"Sex (Male) × Surname (Same)"),

Estimate = NA,

SE = NA,

'p-value' = NA))

lmm_res[lmm_res$sex_student == "Female", c(3:5)] <-

round(summary_female$coefficients[c(2:4), c("Estimate", "Std. Error", "Pr(>|t|)")], 3)

lmm_res[lmm_res$sex_student == "Male", c(3:5)] <-

round(summary_male$coefficients[c(2:4), c("Estimate", "Std. Error", "Pr(>|t|)")], 3)

lmm_res %>%

gt(groupname_col = "sex_student") # 500*350

# Figure. S1 ----------------------------------------------------------------

fig.s1_pop <- data_census_population_mod %>%

filter(year == 2018) %>%

mutate_at(vars(name_eng), ~ factor(name_eng, levels = factor(.))) %>%

pivot_longer(cols = c(male, female), names_to = "sex", values_to = "n") %>%

ggplot() +

geom_bar(aes(x = name_eng, y = n, fill = sex), stat = "identity", color = "black", position = "dodge") +

scale_fill_manual(values = c("gray", "white"), name = "",

labels = c("Female", "Male")) +

scale_y_continuous(breaks = c(0, 500000, 1000000), labels = c("0", "500000", "1000000")) +

labs(y = "Population", title = "(a)") +

theme_bw(base_size = 15) +

theme(panel.grid = element_blank(),

axis.text.x = element_text(angle = 90, hjust = 1, color = "black"), axis.title.x = element_blank(),

axis.text.y = element_text(color = "black"),

legend.position = c(0.8, 0.9), legend.background = element_blank()) ; fig.s1_pop

fig.s1_couple <- data_census_couple_mod %>%

filter(year == 2018) %>%

arrange(desc(number_couple)) %>%

mutate(couple_eng = paste(name_eng1, name_eng2, sep = "-")) %>%

mutate_at(vars(couple_eng), ~ factor(couple_eng, levels = factor(.))) %>%

ggplot() +

geom_bar(aes(x = couple_eng, y = number_couple), stat = "identity", fill = "gray", color = "black") +

labs(y = "Number of couples", title = "(b)") +

theme_bw(base_size = 15) +

theme(panel.grid = element_blank(),

axis.text.x = element_text(angle = 90, hjust = 1, color = "black"), axis.title.x = element_blank(),

axis.text.y = element_text(color = "black")) ; fig.s1_couple

fig.s1_pop + fig.s1_couple + plot_layout(widths = c(1, 1.5)) # 1200 * 600
